# Novel Reflections on the Autonomy and Responsibility of Science

**DOI:** 10.3389/frma.2021.615357

**Published:** 2021-07-05

**Authors:** Fabian Hempel

**Affiliations:** ^1^ Institute of Sociology and Economics, Department of Social Sciences and Public Affairs, Bundeswehr University Munich, Munich, Germany; ^2^ Working Group on Sociological Theory, Social Differentiation and Governance, Department of Theoretical and Normative Foundations, SOCIUM Research Center on Inequality and Social Policy, University of Bremen, Bremen, Germany

**Keywords:** autonomy of science, responsibility of science, science in fiction, science in society, modern society, sociology of science, sociology of literature, modernity

## Abstract

This paper explores how cultural understandings of the autonomy and responsibility of science in modern society are manifested in two contemporary science novels about research misconduct in biomedical research. In doing so, it looks at several facets of the societal impact of and on public and private biomedical research, especially with respect to changing authority relations and their epistemic and institutional consequences. The analysis focuses on the multi-layered ways in which social and epistemic interests are treated in Allegra Goodman’s *Intuition* and Jennifer Rohn’s *The Honest Look*. Goodman’s novel demonstrates how, intensified by the economization of science, internal cultural and institutional aspects of the scientific field enable social configurations that, among others, encourage scientific malpractice and lead to the delay of research projects epistemically and socially worth pursuing. In contrast, Rohn’s novel exemplifies the corrosion of the ideal scientific ethos by profit-driven practices in private-sector biomedical sciences. The concluding discussion juxtaposes these findings with pertinent contemporary phenomena in modern science systems to provide a more substantial understanding of the interpenetration between science and other social spheres.

## Introduction

The social institutions of science are inherently linked to the concept of modernity, as it is the advanced functional differentiation of modern societies that has enabled the development of science into a distinct social subsystem. While all types of social communities can and do spawn institutions that generate theoretical and practical knowledge, modern science and the other institutions of modernity demonstrate a particular sort of co-production (Jasanoff, 2004: 2–3). The social sciences view science and modernity as culturally and structurally embedded in each other, and society at large has considered them the paragons of ambivalent progress–at least from the European Enlightenment to the present era of climate change and pandemics (Wagner, 2016: 23; Renn, 2020: 12). At the same time, they have also been faulted with generating many contemporary environmental hazards and social risks (Beck, 1992: 163; Collins and Pinch, 1993: 1–3).

The organization and practice of scientific research is thus a key element of contemporary knowledge production that both structures and is structured by modern society. Nevertheless, the idea that the institutional and epistemic autonomy of science is a functional imperative has become a foundational notion, and it is supported by the scientific community’s own ideal of science as an effectively democratic, self-correcting system in which the validity and worth of epistemic assertions are based solely on clear intellectual criteria (Merton, 1973; United States Congress, 1981). This ideal of science is often accompanied by the pragmatic and more realistic acknowledgment that science indeed has a social purpose beyond that of certifying knowledge and requires resources from the society at large if it is to operate in a socially beneficial and responsible manner. Scholars have traced the increasing prevalence of partisan societal, political, and economic considerations in the contemporary governance, organization, and practice of science. These include the focus and sources of research funding and economic outcomes of scientific research (Resnik, 2009; Hackett, 2014). These phenomena would seem to contradict the foundational notion of science as an autonomous system with its own field-specific values, organizational modes, and reward structures.

In this paper, I consider the implications of these developments and the cogency of the ideal model of science open to inquiry. Employing the realist science novels *Intuition* (Goodman, 2010) and *The Honest Look* (Rohn, 2010b) as tools for sociological inquiry, I explore cultural understandings of the autonomy and social responsibility of science, with a focus on biomedical research and the epistemic and institutional consequences of changing authority relationships. It is the methodological starting point of this paper that literary imaginations are the product of cultural perceptions and that science novels may convey conceptual insights by centering their stories on social actors and constellation, and on interactions both within and beyond the institutions of science (Engelhardt and Hoydis, 2019; Gaines et al., 2021).

The next sections provide a theoretical background and an overview of discourse on scientific autonomy and responsibility; establish a methodological foundation for employing fiction as an epistemic tool for the social studies of science; perform in-depth readings of the two novels; and discuss those readings in light of scholarly observations of contemporary science systems.

## Conceptual Background

Several strands of social and political theory provide the sociological underpinnings for my analysis of cultural understandings of scientific autonomy and responsibility: the duality of science and society in the evolution of modernity, and theories of social autonomy and responsibility.

### Science and Modernity

Scholarly use of the term modernity varies widely, but at its conceptual core is the ongoing interplay between new and traditional cultural and institutional patterns (Münch, 1986: 11–34; Bhambra, 2015: 692). An idealistic understanding associates modernity with a commitment to the inalienable freedom of human beings and trust in their capacity to reason. According to Peter Wagner, “this basic commitment translates into principles of individual and collective self-determination and in the expectation of ever-increasing mastery of nature and ever more reasonable interaction between human beings” (Wagner, 2012: 4). Modern society’s prevalent structures and processes arise from these principles and expectations. This involves an ongoing functional differentiation into a set of largely self-contained social subsystems with institutionalized means of social production, social roles, and cultural value spheres (Schimank, 2015a: 392; Schimank, 2015b: 415). The formation of modern science systems is a paradigmatic example of functional differentiation with its inclination to produce autonomous actors and the individual and collective mastery of a set of tangible and intangible assets (Wagner, 2008: 98).

Under the influence of functional differentiation, science has evolved into a highly productive social institution with the prerogative to produce, advance, and certify knowledge (Merton, 1973: 270). It is modern society’s principal means of acquiring and applying new knowledge that is of epistemic, and, if applicable and socially desirable, of practical interest. In hindsight, scientific practices and scientific knowledge have substantially increased modern society’s capacity for action (Adolf and Stehr, 2016: 42) and fostered its belief in the intentional controllability of progress and (Schimank, 2014: 118–123) and in its ability to remove all those aspects of the social and natural world that are constitutively unavailable (Rosa, 2020: 86–101). Science plays a societal role that cannot be filled by any other social subsystem (Parsons and Platt, 1973: 53–57), but it is also structurally dependent on resources produced by other societal subsystems (Luhmann, 2013: 113–114).

A key feature of modern societal subsystems is the cultural valuation and institutional enactment of individual and collective autonomy, whereby social actors allegedly operate by self-referential, field-specific value systems that are not derived from or determined by external sources of authority (Luhmann, 2013: 108–110). Different normative and instrumental notions of the autonomy and social responsibility of scientific research have been part of its institutional and governance discourses at least since World War I (Kaldewey, 2013: 17–23; Stilgoe and Guston, 2017: 853–857). The American Association for the Advancement of Science’s Statement on Scientific Freedom and Responsibility is a prototypical example of the scientific community’s perception that both scientific autonomy and social responsibility are integral to the production and application of knowledge: “Scientific freedom is the freedom to engage in scientific inquiry, pursue and apply knowledge, and communicate openly (…) Scientific responsibility is the duty to conduct and apply science with integrity, in the interest of humanity, in a spirit of stewardship for the environment, and with respect for human rights” (as cited in Jarvis, 2017: 462). Concepts of social autonomy and responsibility are thus key to understanding the ways that the science system and the wider society shape each other.

### Autonomy and Responsibility of Science

Immanuel Kant’s conception of autonomy and heteronomy provides an ideal starting point for understanding both terms as analytical micro-, meso-, macro-level variables in science and society. Following Kant [Kant, 2011 (1785): 109–111], heteronomous acts are those determined by the will of entities other than their perpetrators. And purely autonomous acts are those determined solely by the will of the actor. Pure autonomy requires actors without external links. Empirically, no social action is purely autonomous. Social action is always determined by a combination of both internal and external factors. But Kant’s juxtaposition can serve as a reference frame for social actions, with the conditions for autonomy being satisfied whenever social actors employ self-reflexive procedures in the process of determining whether and how to act (Dworkin, 1988: 20).

Isaiah Berlin’s differentiation between inner and exterior freedom connects the notion of the autonomy of action with the concept of social responsibility (Berlin, 2005: 20). Berlin imagines inner freedom as a retreat to an internal citadel–an area free of outside forces that allows for autonomous action without interference by others–whereas exterior freedom pertains to the capacity of actors to control actions whose consequences extend to other actors. This actor-focused perspective leads to a sociological understanding of autonomy and responsibility within the framework of action theory, which aims to explain how and why different types of social protagonists act in the ways they do (Kalter, 2015: 75). In action theory, the underlying mechanisms of action are analyzed on the micro level, with a focus on specific interactional contexts and actor constellations. These situational analyses lay the foundation for meso- or macro-scale explanations.

Action theory is based on the notion that sociality is continually produced and reproduced by the interplay between social actions and social structures (Schimank, 2015b: 415–416; Schimank, 2016: 16–27). Social actors never fully determine the genesis and outcome of their own actions due to their inclusion into constraining or enabling social structures and relationships. Autonomy is defined as a social actor’s degree of control over setting and approaching objectives (Gläser and Schimank, 2014: 47; Gläser et al., 2020: 5). Purposive social actions–acts that are in some part oriented toward others and involve a choice and the evaluation of multiple alternatives–are social precisely because they might have intended or unintended effects on other social actors (Merton, 1936: 895; Offe, 1989: 758). A social actor shares responsibility for such purposive actions, thus linking the social action to the autonomy and responsibility of the actor.

We can analyze the generally accepted institutional goals of science in terms of this understanding of autonomy and social responsibility. The autonomy of science from other social subsystems might then be measured in terms of the relative impacts of scientific and non-scientific actors on both the epistemic and institutional means to produce and certify knowledge. Though it is analytically difficult to separate purely epistemic from institutional actions, it is useful to differentiate between epistemic and institutional autonomy, if only heuristically, e.g. by compartmentalizing scientific capital into the purely epistemic and purely social (Bourdieu, 1991: 7).

The epistemic and social consequences of modern science systems are closely connected to systems for technological innovation and production (Genus and Stirling, 2018: 63). It is difficult to control the intents and impacts of a scientific action, and “during its early stages, when it can be controlled, not enough can be known about its harmful social consequences to warrant controlling its development; but by the time these consequences are apparent, control has become costly and slow” (Collingridge, 1980: 19). Notwithstanding, if these consequences–whether intended or unintended, beneficial, or harmful–are the outcome of a linked chain of action, all actors involved in the planning and implementation of those actions must bear some degree of responsibility. Heather E. Douglas (Douglas, 2003: 63–66) distinguishes scientists’ general moral responsibilities as members of society from those that are specific to their roles in defining and performing scientific research and related actions. This distinction applies to both individual and collective actors. Assigning responsibility for the intended and unintended consequences of social action highlights the structural tension between scientific autonomy and responsibility.

In scholarly and policy discourse, arguments about the various social forces that support or constrain autonomy and responsibility in the organization and practice of scientific research focus on three basic topics (Wilholt and Glimell, 2011: 352–357; Wilholt, 2012: 11–12):

• Freedom of research: The autonomy of science is a prerequisite for free inquiry, which is the most effective way of organizing and conducting research that is primarily motivated by purely epistemic interests (Polanyi, 1945: 142). The productivity of scientific research is essential to the function of other social spheres, as scientific knowledge is required for informed decision-making, particularly in the government policy and economic spheres (Stehr, 2015: 108).
• Accountability: Because of its potential impacts and systemic openness, science is inseparable from society and requires societal accountability. The interests and actions of individual and collective actors within science are not the result of purely epistemic considerations but of interactions with other actors both within and beyond science: they therefore require outside oversight.
• Targeted research: Modern society requires science to contribute to its context- and time-specific social needs. Basic research that derives from purely epistemic considerations cannot be expected to generate enough socially applicable knowledge to meet these demands, so science must be strategically organized and governed (Gibbons, 1999: C84).

These arguments about the independence and accountability of scientific inquiry pertain to all actors in the science system, including individual scientists, research groups, private and public research institutes, universities, and funding organizations. At their heart, lies the classical scientific ethos-norms of conduct such as universalism, disinterestedness, communalism, and organized skepticism that “are in varying degrees internalized by the scientist, thus fashioning his scientific conscience” (Merton 1973: 269) and the notion that the balance between autonomy and responsibility impacts the production and, ultimately, the advancement of knowledge (Kaldewey, 2013: 410). For this reason, and because of their rhetorical efficacy, “scientific autonomy” and “scientific responsibility” have joined the everyday parlance of both scientists and policymakers (Panofsky, 2010: 140).

## Methodological Background

Contemporary literature’s increasing engagement with modern science (Gaines, 2001; Rohn, 2010a) and distinct observational qualities make it a fruitful resource for social studies of science. Cultural studies scholars, sociologists, and literary theorists have delved into the nexus between understandings of social interaction and the creation and consumption of literature. Rita Felski notes that “reading involves a logic of recognition; that esthetic experience has analogies with enchantment in a supposedly disenchanted age; that literature creates distinctive configurations of social knowledge; that we may value the experience of being shocked by what we read” (2008: 14). Erkki Sevänen maintains that fiction can process and represent societal developments; he regards modern literature “as communicative acts between authors and society” (2018: 53). Similarly, Albrecht Koschorke (Koschorke, 2018: 51) considers narration a communicative game with hypothetical problem-solving possibilities that processes and resolves topics that matter to the narrators and their intended audience. A sociology of knowledge approach to literature acknowledges that social actors such as authors, general readers, and scholars have internalized tacit and explicit knowledge of the social worlds they are embedded in and of these worlds’ social and cultural conventions, attitudes, and rules (Sevänen, 2018: 52). Literary and cultural studies scholars have long plumbed literature and other cultural artifacts for their latent social analysis and knowledge in understanding postcolonial societies (Ashcroft, 2017), economic history (Roxburgh, 2015), or contemporary financial capitalism (Vogl, 2014).

The disciplinary division of labor between literature and the social sciences, even while addressing similar topics of inquiry, has been an issue since the mid-nineteenth century when scholars “contested with one another the claim to offer the key orientation for modern civilization” (Lepenies, 1992: 1). In Europe in particular sociology established itself as a distinct epistemic culture that was situated between the natural sciences and the humanities. Large swathes of the social sciences in France, England, and Germany underwent a process of conceptual and methodological scientification that separated them from their literary counterparts. In North America, the assumption that the analysis of sociological topics could be advanced by exploring fictional literature was more established, especially in the Chicago School of Sociology (Coser, 1972: 2–3). For instance, Florian Znaniecki (Znaniecki, 1934: 193–197) advanced a methodology in which observations of the social by other social actors–what Niklas Luhmann calls first-order observations (2013: 224–225)-constitute a form of sociological utilizable data. Znaniecki assigned literature to this class of data on human experience (Znaniecki, 1952: 134), because it is composed by authors who process the cultural tendencies and social interactions of the societies they live and work in. This line of reasoning anticipated the general approach employed by the sociology of literature and literary and cultural studies to substantiate the value of fictional literature for scholarly social analysis (Becker, 2007: 5; Matthies, 2016: 17; Farzin, 2019: 140; Váňa, 2020: 184–186).

Fictional literature allows the reader to experience particular social worlds from the inside (Felski, 2008: 92). Fiction does not necessarily provide documentary representations of the social world, but rather “what-if” constructions of the interaction between social actors who deliberately process, embed, and configure a variety of themes, events, and relationships. Fictional texts offer a means “with which to probe into reality, testing certain features of the world as described in the text” (Longo, 2015: 140) precisely because they can highlight the cultural desiderata of particular social milieus and discursively shared cultural conceptions. These observations form the basis for my analysis of science novels, which adopts Helmut Kuzmics’ and Gerald Mozetič’s three premises for the use of literary sources in sociological analysis (Kuzmics and Mozetič, 2003: 26–35): First, fiction can illustrate sociologically relevant themes and phenomena. Second, literature has the potential to be a descriptive source of cultural and social representations. Third, fictional texts can bear latent and/or manifest explanatory potential of social phenomena.

The novels I examine fall within a category of fiction–variously known as science novels, lab lit, and science in fiction–that features researchers as main characters and explores scientific problems, research practices, and their respective organizational and cultural contexts (Haynes, 2016a: 128–130; Pilkington, 2019: 1–2; Gaines et al., 2021). These works of fiction enable examination of the purposive actions of scientists and “the way in which that fictionalized process is affected by the author’s reconstruction of the dominant discourse of the day, both within and beyond the scientific community” (Schaffeld, 2016: 121). Following on Znaniecki and Luhmann, I consider my analysis of these novels a second-order observation of science in society (Gaines et al., 2013: 9). While sociologists can and do produce first-order observations of the culture, organization, and practice of science, such observations are limited by the methodological constraints of conventional research. Novelists, on the other hand, are more autonomous in their scope to observe, participate in, respond to, and imagine “what-ifs,” creating a singular configuration of social circumstances for the sociologist reader to process alongside first-order empirical accounts and sociological survey data.

## Analysis of Two Science Novels

Summarizing the above considerations, the conceptual and methodological premises of my analysis are threefold: First, the autonomy and responsibility of social actors are distinct institutional and cultural features of modern society. Second, the autonomy and social responsibility of science are central cultural frames in modern science systems, though their institutional manifestations and effects may vary considerably. Third, science in fiction is the literary product of culturally situated social practices that observe, process, and display cultural understandings of science in society. I employ a form of qualitative content analysis with a textual and thematic focus that is guided by two basic research questions (Braun and Clarke, 2006; Schreier, 2017): What understandings of the autonomy and societal responsibility of science are displayed in the novels? How do they correspond with actual cultural and scholarly conceptions of scientific autonomy and responsibility? Progressing from a descriptive summary to a thematic interpretation of the text, I do not aim for a generic account of autonomy and responsibility, but rather to “elicit meaning, gain understanding, and develop empirical knowledge” (Bowen, 2009: 27) of a set of individual and novel cultural understandings.

Allegra Goodman’s *Intuition* (2010) and Jennifer Rohn’s *The Honest Look* (2010b) are set in biomedical research laboratories in the United States. and Europe respectively. Literary scholars and critics have called both novels “lab lit,” a term Jennifer Rohn herself coined to describe decidedly “realistic novels that contain scientists as central characters plying their trade” (Rohn, 2010a: 552; Pilkington, 2019: 301). Lab lit typically details everyday laboratory life, engages “with the process of ‘doing science’,” and indicates “realistically how actual scientists think and behave in the intense atmosphere of a research laboratory” (Haynes 2016a: 36). Both *Intuition* and *The Honest Look* derive from the authors’ immersion in contemporary science systems and local research sites. In order to observe the inner working of research laboratories for her novel, Goodman did a considerable amount of field observation at the Whitehead Institute for Biomedical Research in Cambridge, Massachusetts (Longhito, 2007: 2,272). Jennifer Rohn, besides being a novelist and science journalist, works as a cell biologist at University College London and leads her own research lab (Rohn, 2008: ix). Moreover, scientists who have reviewed *Intuition* (Thomas, 2006: 1,235) and *The Honest Look* (Herndon, 2010: 1,039) describe their stories as plausible, credible, and thematically relevant to current developments in modern science systems. Both novels depict prototypical scenarios of the organization and practice of public and private biomedical research and are thus well-situated to explore the autonomy and social responsibility of science.

### An Intuitive Look at Public Biomedical Research

The story of Goodman’s *Intuition* revolves around the fictional Philpott Institute, a publicly funded biomedical research laboratory in Cambridge, MA. The Philpott is not affiliated with the local universities, Harvard and the Massachusetts Institute of Technology, but it is trapped in competition with them for research funding and peer recognition. The laboratory staff comprises the Philpott directors, Sandy Glass and Marion Mendelssohn, several postdoctoral researchers–including Cliff Banneker and Robin Decker–and various lab technicians and graduate students. Shortly before the end of Banneker’s postdoctoral contract, after years of modest or disappointing research results, he suddenly seems to have made crucial progress in the development of R-7, a modified virus that has been designed to transform cancer cells into normal cells. After his newly gathered data hints that R-7 might be able to eradicate cancer tumors from mice, Glass and Mendelssohn concentrate all the lab’s resources on follow-up research. These efforts result in the rushed publication of a much-anticipated research article in the prominent interdisciplinary science journal, Nature, and are touted as a major scientific breakthrough in the news media. Banneker’s data is the basis for a successful grant proposal at the National Institutes of Health (NIH), the United States’ largest biomedical research agency and source of public funding.

In order to verify the data and concomitant conclusions, Mendelssohn and Glass assign Decker, a post-doc whose own project shows relatively little promise, the task of reproducing Banneker’s work *in vivo*. Her attempts fail several times and, after doubting her own research abilities, she begins to suspect that something might be wrong with Banneker’s initial data. Decker discovers inconsistencies in Banneker’s recordkeeping and adherence to experimental protocol–potentially a form of misconduct in and of itself–but it is her growing intuition that the data was intentionally manipulated that leads her to inform Mendelssohn and Glass of her suspicions. When an internal review exonerates Banneker of research misconduct, Decker becomes a whistleblower for NIH’s Office for Research Integrity in Science, which oversees the probity of federally funded research activities. This results in a scientific controversy, and the Subcommittee on Science and Technology of the United States House of Representatives summons the involved members of the lab to appear at a series of public hearings. Meanwhile, further observations of R-7’s effectiveness at the Philpott lab show that the initially reduced tumor cells in mice have begun to reappear. Though this is an intriguing finding in itself, failure to reproduce Banneker’s results and growing internal doubts, as well as the negative publicity and external pressure, prompt Glass and Mendelssohn to retract the Nature paper. Other laboratories are also unable to fully reproduce Banneker’s findings, and attention soon turns away from R-7 as a cancer treatment. The misconduct case is, however, dismissed on procedural and political grounds, when Decker’s reputation is thrown into question.

*Intuition* offers a “what-if” narrative of research misconduct and “details the factors that allow an insufficiently substantiated claim to gain credence, however transitorily, in the scientific community” (Kirchhofer and Roxburgh, 2016: 159). It can be read as a critique of how scientific reward systems based on competition, originality, and positive results–exacerbated by funding pressures and the reward structures of individual and collective research careers–can interfere with norms of scientific responsibility and foster poor practices, outright misconduct, and, ultimately, false data and conclusions (Kalleberg, 2015: 313–314). The novel leaves open the question of whether Banneker’s unreproducible research findings are the intended or unintended consequences of questionable research practices or outright scientific misconduct: “Perhaps his work with R-7 had been more about ideas than concrete facts; perhaps his findings had been intuitive rather than entirely empirical. He had not followed every rule” (Goodman, 2010: 320). In this multi-layered novel, the characters and research organizations are so entangled and embedded in various social configurations–postdocs, research group, directors, the Philpott Institute, other scientific and political institutions, and media–that they are never entirely in control of their own actions, let alone the outcomes.

Though this lack of autonomy affects the work of the postdoctoral researchers most acutely, it also constrains the actions of the lab directors and limits the capacity of the allegedly independent research institution at the heart of the novel:

Two to a bench, like cooks crammed into a restaurant kitchen, the postdocs were extracting DNA in solution, examining cells, washing cells with chemicals, bursting cells open, changing cells forever by inserting new genetic material. (…)
In 1985, the Philpott was famous, but it was full of old instruments. Dials and needle indicators looked like stereo components from the early sixties. The centrifuge, designed for spinning down cells in solution, was clunky as an ancient washing machine. There wasn’t enough money to buy new equipment. There was scarcely enough to pay the postdocs (Goodman, 2010: 3–4).

From the perspective of the postdoctoral researchers, the laboratory, indeed the institution of science in general, functions like a sort of prison workshop: “Years and years of manual labor went by. New results filtered through only on the rarest occasions, and always to other people. Miracles didn’t happen, but Cliff and his friends kept on working. Like scientific sharecroppers, they slaved all day. They were too highly trained to stop. Overeducated for other work, they kept repeating their experiments. They kept trying to live on their seventeen-thousand-dollar salaries” (Goodman, 2010: 20). This realization comes to Banneker in the midst of a knowledge production crisis that threatens to undermine his hopes of ever obtaining a permanent job as a research scientist: After developing and testing the R-7 variant for two years, he has found no evidence of its effectiveness in reducing cancerous tumors. Decker’s project-“an analysis of frozen samples of blood, collected over the years from cancer patients who had died of various forms of the disease” (Goodman, 2010: 7) in search of a unifying syndrome underlying their diverse conditions–has been similarly fruitless and short of positive results.

Both from an epistemic and institutional standpoint, the ideal laboratory has been imagined as an inner citadel that locks out all aspects of the natural and social world that defy control so that such research sites “not only improve upon natural orders, but (…) also upgrade social orders” (Knorr-Cetina, 1999: 28) of laboratory processes and research organization to become an enhancing instrument of scientific work. But Banneker, Decker, Glass, and Mendelssohn are not acting within such an epistemically unconstrained structure. External social forces and expectations constantly shape their activities. The postdocs are especially dependent on the tangible output of useful, publishable results to establish their reputations within the biomedical research community. The novel depicts a classical and still prevalent scientific reward structure that emphasizes originality in its various forms–new discoveries and paradigm-shifting breakthroughs–which causes “extreme inequality with regard to scientific productivity and the awarding of priority” (Stephan, 1996: 1,203). When Banneker’s experiments are unsuccessful, Glass and Mendelssohn order him to abandon his hypothesis and work to support the lab’s other ongoing projects. And when he defies their orders and runs a final set of experiments that suddenly indicate that R-7 might be able stop or even reverse cancer growth, they reverse gears and order the rest of the lab to shift their attention to Banneker’s project. Though they admonish the post-docs that “[t]there is no such thing as your own project in this lab” (Goodman, 2010: 6), it is abundantly clear that, like Banneker, Robin and the other post-docs depend on the success of their own independent research ideas to demonstrate their ability to produce original scientific insights and insure their futures as research scientists.

Both epistemically and institutionally, control of goal definition and achievement is severely restricted for graduate students, postdocs, and early career researchers: “On the ground, in the lab, intuition was a restricted substance. Like imagination and emotion, intuition misled researchers, leading to willful interpretations. While scientists like Mendelssohn knew how to wield it properly, young researchers had their intuition tamped down lest, like the sorcerer’s apprentice, they flood the lab with their conceits” (Goodman, 2010: 183). Thus, the novel presents the lab as a collective workshop in which research insights are “appropriated by the managers without further ado” (Zwart, 2017: 184). Its powerless postdocs have internalized the rules of the game whereupon scientific rewards and reputations are built on individual and not necessarily on collective accomplishments. In that sense, the laboratory resembles a feudal community: “There are the lords and ladies like Glass and Mendelssohn, and then the postdocs are the vassals paying tribute every year in the form of publications, blood, sweat, tears, et cetera” (Goodman, 2010: 211).

Yet the story illustrates in several events that Glass and Mendelssohn, reminiscent of actual feudal lords in medieval Europe, are far from omnipotent. Instead, their work, reputation, and positions equally depend on the output of postdocs and, in consequence, on successful grant applications in order to organize subsequent research in their laboratory. The lab is part of a non-university research institute that is presented as a “poor principality” (Goodman, 2010: 109), “has run a deficit for the past three years” (Goodman, 2010: 290), has no substantial institutional funding, and relies therefore on the ability of its constituent research groups to continuously attract research funds. It is “governed by strict Darwinian principles. Investigators broke even or went bankrupt, losing staff and space and equipment to their rivals (…) Lab directors without funding had little recourse; they took desperate measures: they switched fields, or retired, or sometimes left science altogether” (Goodman, 2010: 17). While simultaneously disagreeing on the exact way to proceed with Banneker’s research, Glass and Mendelssohn agree to establish strong priority claims with regard to the potential results of the R-7 experiments in order to substantiate their grant proposal that could provide research funding for several years (Goodman, 2010: 71). Both acknowledge the dire economic situation of the lab that can only be overcome by eventually overplaying the classical reward game and rushing ahead with the publication of inconclusive results before someone else can stake similar claims. In hindsight, their prediction becomes a self-fulfilling prophecy. The lab’s concentration on R-7 misspends its limited material and personal resources and reproduces poor research.

To sum up, *Intuition* exemplifies a science system in which the epistemic and institutional autonomy of postdocs, senior researchers, and independent research institutes is severely constrained by internal and external factors. That rationalizes questionable research practices, unintentional negligence, or even intentional misconduct. While the internal reward structure can be considered as a historically and contextually contingent outcome of internal developments within the social system of science, the lack of institutional funding and the dependence on project-based research grants are decidedly external social forces that foster the structural importance of priority claims, peer-acknowledged reputations, and swift publications of research findings. These phenomena are, in the novel and in most if not all modern research systems, rooted in the economization of modern society and modern science in particular (Schimank, 2021: 148–149). Moreover, bad and fraudulent science is epistemically and socially useless, even harmful, and a waste of resources. Apart from detrimental financial implications, such poor and misleading research practices as depicted in the novel curb the progress of the biomedical sciences (Chevassus-au-Louis, 2019: 105–114). It is therefore not far-fetched for society at large to develop doubts with respect to the social responsibility and surplus value of contemporary research, especially with those projects that are funded by public bodies (Schomberg, 2013: 63).

At the mentioned congressional hearing a congressman attacks Glass, Mendelssohn, and their lab by claiming that science is “corrupted by (their) desire for more and still more funding, and a lust for quick results” and rewards “intellectual dishonesty” (Goodman, 2010: 269). While this is in part factual and an unintended consequence of established funding and reward structures, this is not entirely the result of internal processes within public research systems. The novel latently alludes several times to science policy paradigms that prioritize economic considerations for financial costs and the collective organization of research analogous to market competition, such as New Public Management or other forms of academic capitalism (Mirowski and Sent, 2008: 637; Berman, 2014: 420; Münch, 2014: 1–12; Schimank and Volkmann, 2017: 175; Jessop, 2018: 105). Accordingly, several characters in the novel–for instance the congressman who lamented what could be called the corrosion of the ideal scientific ethos (Sennett, 1999; Goodman, 2010: 262–263) emphasize that science should be used to improve the economy, the most important subsystem of modern society. Considering the tension between and the ambivalent impact of these internal and economic constraints on the governance of public research and the production of scientific knowledge, it remains to be seen how the ideal-typical imperative for the autonomy and social responsibility of science is manifested in cultural representations of science that look upon private and deliberately commercial biomedical research, such as with the novel discussed in the next section.

### An Honest Look at Private Biomedical Research

Jennifer Rohn’s *The Honest Look* (2010b) explores the early stages of Claire Cyrus’s research career in a pharmaceutical research laboratory of a private company, the scientific and economic pressure to produce ground-breaking and assetizable treatments, and the vested interests of the corporate biomedical sciences (Haynes, 2016b: 36). Set in the Netherlands, the story features Cyrus as one of the few researchers in the world who can operate the so-called Interactrex 3000, “a must-have tool for those dedicated to finding cures for the killer diseases that have plagued mankind for centuries” (Rohn, 2010b: 2). This expensive machine, christened by her as Raison D'être (sic), “can peer into living cells and watch proteins interact in real time” (Herndon, 2010: 1,039). Having been trained by Maxwell Bennett, a renowned biologist, inventor of the Raison, and her former PhD supervisor at the University of Liverpool, she is successfully headhunted by Stanley Fischer, the CEO of NeuroSys, a biotech startup in the metropolitan area of Amsterdam. Because of the machine’s potential significance for advancing the company’s research into Alzheimer’s disease, Cyrus begins to collaborate, among others, with Allan Fallengale, “a much older lecherous senior scientist” (Chester, 2011: 2,936), in order to check the effectiveness of the company’s essential scientific asset, a potential drug for Alzheimer’s called the Zapper.

Much more than *Intuition*, the novel presents the tangible scientific apparatus as “both a character and a foil for the main actant” (Pilkington, 2017: 301) to the effect that Cyrus’s research produces remarkable findings from a purely epistemic standpoint that bears problematic implications for the biomedical potential and financial stability of the company. Machines and engineering techniques like the Interactrex are the eyes and backbone of modern biomedical research and underpin its capacity for scientific insight and pharmaceutical applicability. Hence, the story illustrates how the development, existence, and access to machines underpins the autonomy of research practices:

The Raison still threw spectacular fits, but she was getting better at dealing with the machine. And the experiments were finally starting to work. She found being an expert at something so much more gratifying than her lowly PhD student experience. The Raison wasn’t some prototype, cobbled together with gaffer tape and aluminum foil into a massive rattling thing that the rest of the department laughed at behind her back. It was a gleaming state-of-the-art machine, and the whole world was watching its first paces to see how it fared (Rohn, 2010b: 46).

*NeuroSys*, “a one-trick pony” (Rohn, 2010b: 12), has high expectations on the Zapper. Its corporate success depends on the treatment’s practical capability and on the validity of the so-called Universal Aggregation Principle, its underlying “revolutionary theory explaining the pathology behind Alzheimer’s disease” (Rohn, 2010b: 24). This method underpins the pure scientific, institutional, and economic capital of NeuroSys and its senior scientists:

NeuroSys had been founded ten years previously to develop treatments for neurodegenerative diseases such as Alzheimer’s. Its scientists, headed by Alan Fallengale and Ramon Ortega, had discovered a key vulnerability underlying these disorders and designed the company’s first key drug: a compound called NS158, otherwise known as The Zapper. Patents were filed, NeuroSys was floated on the stock market, and it was rumored that patient trials were just around the corner. Emboldened by these successes, the company was expanding into other disorders, including stroke, and had recently convinced venture capitalists to fund their next phase. Hence, the purchase of the Interactrex 3000 and the hiring of Claire (Rohn, 2010b: 13).

Being new on the job, a junior scientist with insignificant clout, and the only one in the company capable of using the Interactrex, Cyrus is exclusively assigned to modify and improve the Zapper. Up to this point, due to her unique skill in operating the machine, her scientific independence is not constrained at all, at least from a purely epistemic point of view with respect to this particular task. Intentionally provided by the company’s leading researchers to fully utilize the potential of the machine, this organizational configuration allows her to conduct comparatively autonomous research and collaborate almost on a peer-to-peer level with scientific and administrative superiors. Serendipitously, she discovers a fatal flaw in the underlying Universal Aggregation Principle that explains both the insufficient impact and a detrimental side effect of the drug: “Experiment, result, interpretation, the three links in the chain of this tidy, ordered profession that had seduced her. A conveyor belt of logic that only flowed in one direction, if you set up your experiments properly. No Universal Aggregation, no target for the Zapper: no cure for Alzheimer’s” (Rohn, 2010b: 99). Based on Cyrus’ findings, Joshua Pelinore, the company’s leading bioinformatician, predicts “the drug in its current form (…) to seriously impair higher cognition in healthy brain cells–it may cause more problems than the Alzheimer’s it’s trying to cure” (Rohn, 2010b: 240).

Cyrus and her superiors know what would happen to NeuroSys if its scientific and economic mainstay turned out to be flawed: Investors would lose trust in the firm’s potential and cease additional funding. Subsequently, the company would go bankrupt due to a lack of alternative revenue streams. “Nothing else we're working on is even close to being marketable” (Rohn, 2010b: 131). Over the further course of the story, Cyrus reluctantly informs her peers, the lab’s superiors, and the company’s management for “it was one thing to react reasonably about a potential flaw that might make necessary a minor chemical adjustment in an established drug (…) But it would be quite another to be faced with the destruction of a life work, a cherished theory and the entire reason for NeuroSys’s existence” (Rohn, 2010b: 190–191). In reaction to this negative scientific breakthrough that dooms the corporate prospects of NeuroSys and threatens his individual scientific reputation and financial rewards, Fallengale, without consulting his longtime collaborators in the lab and the firm, sells the Interactrex to remove incriminating evidence and to prevent others from reproducing Cyrus’s results. These actions enable him to collect his contractually secured milestone payment. He subsequently resigns from his position within the firm, long before anyone outside can find out the truth (Rohn, 2010b: 308).

In sum, the novel narrativizes three cases of scientific and institutional failures that encompass poor scientific practices and misconduct in private biomedical research. First, the practical utility of the treatment and the epistemic utility of the underlying theory turn out to be wrong, but it remains unclear how much resources were invested by the company to verify the validity of both by additional internal and external research. Second, Cyrus, because of her awareness of the potential personal and institutional implications of the Raison’s findings, initially refrains from telling the truth. Third, Fallengale’s reactions and his successful attempt to remove potentially incriminating evidence constitutes clear intentional scientific misconduct. In that sense, the novel may be read as a counterintuitive argument against an overload of individual and organizational autonomy, at least in epistemic terms, which can result in too much protected space and flexibility within the confines of the firm and with regard to the wider biomedical community (Whitley, 2014: 370–371). While Fallengale might have been motivated by heteronomous motives, his protected senior position within the organization and the extensive trust of his peers and collaborators in his integrity allowed him to act in the way he did. The resulting individual and organizational opportunity structures (Eisinger, 1973: 12) enable social irresponsibility toward scientific and corporate insiders and outsiders. The consequences harm not only the involved scientists, the company, or its investors. It also delays the progress of medical research and, in turn, the potential societal return, especially in the form of potential improvements in treating Alzheimer’s, a disease that affects the lives of millions worldwide, and other forms of neurodegeneration.

Additionally, and in contrast to *Intuition*, *The Honest Look* sheds light on the ambivalent aspects of privatizing knowledge. The case of NeuroSys exemplifies the potential epistemic corruption of privatizing scientific insights and the closed practices of restrictive knowledge-control regimes that curb reviews by disciplinary peers (Hilgartner, 2017: 8–11; Sismondo, 2021). It shows how the transformation of knowledge into assets that can be owned, controlled, and capitalized is a salient feature of modern knowledge societies whose economic outlook is increasingly shaped by technoscientific capitalism (Birch and Muniesa, 2020: 19). Such knowledge can only become visible and testable, to a limited and controllable degree, if it appears to be an owned asset, for instance a patent, a machine, or a drug, that substantiates the social, mostly financial interests of the respective institution that controls it. Such research has ceased to be a public good that is, at least epistemically, owned by scientific communities; it is a practice that contradicts the classical scientific norm of communalism, according to which any scientific insight belongs to the whole research community (Merton, 1973: 273). That limits the capability of external actors within and outside of the scientific field to check the validity of these knowledge claims and objects. Thus, the tale of Claire Cyrus alludes to the dysfunctional consequences of dominant economic considerations that emphasize the need to maintain an organizational front that presents the product as valuable and the company as innovative, which supersedes the basic scientific imperative to find errors in data and to double-proof theoretical and empirical insights (Schimank, 2021: 162).

## Discussion

*Intuition* and *The Honest Look* display a cultural understanding of the autonomy and social responsibility as manifest and latent normative ideals in modern science systems that are insufficiently implemented due to internal and external social constraints on the organization and practice of public and private biomedical research. While each novel portrays the economization of science as a serious impediment to scientific progress, *Intuition* depicts how economic considerations accelerate the classical reward structure of science. This limits the institutional and individual autonomy of research, especially for scientific organizations that lack substantial channels of core funding and for junior researchers who are obliged to produce positive, presentable results in order to demonstrate their ability to the scientific community. In contrast, *The Honest Look* shows how profit-driven research neglects classical research norms by curbing and circumventing the scrutiny of external members of scientific community. Given the biomedical background of the company, none of its depicted members appear to be purposely driven by the prospect of healing diseases or solving epistemic puzzles. Instead, they are culturally, institutionally, and financially more inclined to build a reputation with potential investors, shareholders, and consumers. In turn, the consequences of these different cases are similar: poor scientific practices, intentional and/or unintentional scientific misconduct, and obstacles to scientific and social progress. Taken together, both narratives also allude to the ambivalence of the autonomy and heteronomy of research. Both a lack and an overload of scientific autonomy can lead to socially irresponsible research practices and outcomes.

*Intuition* and *The Honest Look* cannot only be considered as distinct literary outcomes out of a field of cultural production that observes and processes the cultural understanding of science in modern society. From a social studies of science standpoint, both narratives also offer an interpretation of the state and trajectory of contemporary science and modern society’s capacity to identify and adapt to social problems. In this regard, the novels contrast an idealized notion of an autonomous and socially responsible science with research systems that are shaped by diverging internal and external social interests and produce epistemic and social uncertainty. In that sense, their representation of contemporary research resembles that of Kim Stanley Robinson’s *Forty Signs of Rain* (2004). This first part of his *Science in the Capital* trilogy is another literary treatize on, among other things, science in modern society and offers a somewhat grand narrative of the structural constraints internal and external to the scientific field that also applies to Goodman’s and Rohn’s novels. *Forty Signs of Rain* presents a prototypical scenario portraying how and why a modern society, in this case the United States, is currently unable to tackle the grand challenges of human-induced climate change. This is in part due to knowledge and technology gaps, but the main reasons for this insufficient adjustment are socially constructed. Those societal actors advocating for socio-ecological adaptation and mitigation–the main protagonists of the novel taking that stance are researchers working for the National Science Foundation (NSF), the largest federal agency in the United States that funds basic research science and engineering–constantly experience how they do not control the necessary societal positions and do not possess sufficient resources to achieve this societal goal. This is in part due to the inefficacy of modern science systems that is characterized by a divergence between normative and actual cultural and institutional patterns in the organization and practice of research, illustrated by the following quotes:

But science didn’t work like capitalism. That was the rub, that was one of the rubs in the general dysfunction of the world. Capitalism ruled, but money was too simplistic and inadequate a measure of the wealth that science generated. In science, one built up over the course of a career a fund of “scientific credit,” by giving work to the system in a way that could seem altruistic. People remembered what you gave, and later on there were various forms of return on the gift–jobs, labs. In that sense a good investment for the individual, but in the form of a gift to the group. It was the non zero-sum game that prisoners’ dilemma could become if everyone played by the strategies of always generous, or, better, firm but fair. That was one of the things science was–a place that one entered by agreeing to hold to the strategies of cooperation, to maximize the total return of the game.
In theory that was true. It was also the usual troop of primates. There was a lot of tit for tat. Defections happened. Everyone was jockeying for a lab of their own, or any project of their own. As long as that was generating enough income for a comfortable physical existence for oneself and one’s family, then one had reached the optimal human state. Having money beyond that was unnecessary, and usually involved a descent into the world of hassle and stupidity. That was what greed got you. So there was in science a sufficiency of means, and an achievable limit to one’s goals, that kept it tightly aligned with the brain’s deepest savannah values. A scientist wanted the same things out of life as an Australopithecus; and here they were (Robinson, 2004, 133–134).

The characters in *Intuition* and *The Honest Look* experience a similar divergence between idealized norms and actual patterns. In addition to depicting how science works or fails to work, both novels illustrate how epistemic and socially dysfunctional scientific practices (that can lead, for instance, to scientific fraud) are the outcome of two interpenetrating social structures: those that are internal to the scientific community, such as the dissociating effects of classical reward systems and credibility cycles which are grounded in demonstrating scientific priority, producing a lot of fast-paced publications, and securing funding for further research (Braun, 1994: 32–33), and those that are partially or entirely external to the social field of science, notably public and private funding regimes (Chevassus-au-Louis, 2019: 164–175). Both structures constrain the institutional and epistemic autonomy of research and impede the capacity of science to produce insightful and applicable research. In turn, these dysfunctions can and do limit science’s capacity to meet its societal responsibilities as a public good that goes beyond the production of mere economic assets for other social fields (Callon, 1994: 416–418). While a bijective interpretation of the novels, and fictional literature in general, is neither feasible nor desirable, the preceding analysis and this discussion leads to the assertion that through depicting internal and external distortions of the classical scientific ethos, both novels represent literary thought experiments of contemporary research that reinforce and deconstruct the cultural ideal autonomy and social responsibility of science in modern societies.

## Conclusion

In contrast to the ambiguous title of the paper, my reading of *Intuition* and *The Honest Look* has not necessarily produced completely novel insights into the autonomy and social responsibility of contemporary science in modern society. Instead, it has taken the cultural understanding of science as an avenue for a sociological exploration of how these notions are manifested in two salient literary narratives that imagine two different yet comparable tales of research misconduct. Based on the methodological assertion that the narrative depiction of science in contemporary popular culture bears the epistemic potential to offer conceptual insight as it puts social actors, actor constellations, and interactions within and beyond the institution of science at the center stage of their respective stories, the analysis has shown the multi-layered societal impact of and on private and public biomedical research, especially with respect to internal and external authority relations, reward structures, and funding regimes. In this regard, Goodman’s novel emphasizes how internal aspects of the science system, especially its institutional structure and peer-based reward system, enable an ethos that encourages scientific malpractice and subsequently leads to the delay of epistemically more fruitful research projects. In contrast, Rohn’s novel exemplifies the corrosion of the ideal scientific ethos by profit-driven practices in private research organizations. Together, both science novels problematize the ambivalence between scientific autonomy and social responsibility by displaying contemporary dynamics of modern science, notably with regard to the structural pressures due to the increasing economization of public and private research systems.

## Data Availability

The original contributions presented in the study are included in the article/supplementary material, further inquiries can be directed to the corresponding author.

## References

[B1] Adolf M. Stehr N. (2016). Knowledge: Is Knowledge Power?. London, New York: Routledge. 10.4324/9781315543093

[B2] Ashcroft B. (2017). Utopianism in Postcolonial Literatures. London, New York: Routledge.

[B3] Beck U. (1992). Risk Society: Towards a New Modernity. Los Angeles, London, New Delhi, Singapore, Washington DC: SAGE.

[B4] Becker H. S. (2007). “Telling about Society,” in Telling about Society. Editor Becker H. S. (Chicago, London: University of Chicago Press), 2t14.

[B5] Berlin I. (2005). “Two Concepts of Liberty,” in Liberty. Editor Hardy H. (Oxford: Oxford University Press), 166–217.

[B6] Berman E. P. (2014). Not Just Neoliberalism: Economization in US Science and Technology Policy. Sci. Technol. Hum. Values 39, 397–431. 10.1177/0162243913509123

[B7] Bhambra G. K. (2015). “Modernity: History of the Concept,” in International Encyclopedia of the Social & Behavioral Sciences. Editor Wright J. D. (Amsterdam: Elsevier), 15, 692–696. 10.1016/b978-0-08-097086-8.03134-2

[B8] Birch K. Muniesa F. (2020). “Introduction: Assetization and Technoscientific Capitalism,” in Assetization. Editors Birch K. Muniesa F. (Cambridge, Massachusetts: The MIT Press), 1–42.

[B9] Bourdieu P. (1991). The Peculiar History of Scientific Reason. Sociol. Forum 6, 3–26. 10.1007/BF01112725

[B10] Bowen G. A. (2009). Document Analysis as a Qualitative Research Method. Qual. Res. J. 9, 27–40. 10.3316/QRJ0902027

[B11] Braun V. Clarke V. (2006). Using Thematic Analysis in Psychology. Qual. Res. Psychol. 3, 77–101. 10.1191/1478088706qp063oa

[B12] Braun D. (1994). Structure and Dynamics of Health Research and Public Funding: An International Institutional Comparison. Dordrecht, Boston: Kluwer Academic Publishers.

[B13] Callon M. (1994). Is Science a Public Good? Fifth Mullins Lecture, Virginia Polytechnic Institute, 23 March 1993. Sci. Technol. Hum. Values 19, 395–424. 10.1177/016224399401900401

[B14] Chester A. (2011). The Honest Look J. L. Rohn Cold Spring Harbor Laboratory Press, 2011, Pp. 343 ISBN: 978-1-936113-11-8. Proteomics 11, 2936–2937. 10.1002/pmic.201190069

[B15] Chevassus-au-Louis N. (2019). Fraud in the Lab: The High Stakes of Scientific Research. Cambridge, Massachusetts: Harvard University Press. 10.4159/9780674242111

[B16] Collingridge D. (1980). The Social Control of Technology. London: Pinter.

[B17] Collins H. M. Pinch T. J. (1993). The Golem: What You Should Know about Science. Cambridge: Cambridge University Press.

[B18] Coser L. A. (1972). “Introduction,” in Sociology through Literature. Editor Coser L. A. (Englewood Cliffs, New Jersey: Prentice-Hall), 2–7.

[B19] Douglas H. E. (2003). The Moral Responsibilities of Scientists (Tensions between Autonomy and Responsibility). Am. Philos. Q. 40, 59–68.

[B20] Dworkin G. (1988). The Theory and Practice of Autonomy. Cambridge: Cambridge University Press. 10.1017/cbo9780511625206

[B21] Eisinger P. K. (1973). The Conditions of Protest Behavior in American Cities. Am. Polit. Sci. Rev. 67, 11–28. 10.2307/1958525

[B22] Engelhardt N. Hoydis J. (2019). “Introduction: Connectivities between Literature and Science in the Twenty-First Century,” in Representations of Science in Twenty-First-Century Fiction: Human and Temporal Connectivities. Editors Engelhardt N. Hoydis J. (Cham: Palgrave Macmillan), 1–17. 10.1007/978-3-030-19490-1_1

[B23] Farzin S. (2019). “Literatur als Quelle und Methode soziologischer Zeitdiagnose,” in Deutungsmacht von Zeitdiagnosen: Interdisziplinäre Perspektiven. Editors Hastedt H. Depner H. Maaser A. (Bielefeld: Transcript), 137–148. 10.14361/9783839445921-009

[B24] Felski R. (2008). Uses of Literature. Malden, Oxford: Blackwell. 10.1002/9781444302790

[B25] Gaines S. M. Kirchhofer A. Schaffeld N. Schimank U. Weingart P. (2013). Fiction Meets Science: Background and Concept. Bremen: Universität Bremen.

[B26] Gaines S. M. Farzin S. Haynes R. D. (2021). “Introduction:,” in Under the Literary Microscope: Science and Society in the Contemporary Novel. Editors Farzin S. Gaines S. M. Haynes R. D. (University Park, Pennsylvania: Penn State University Press), 1–18. 10.5325/j.ctv1mvw8k2.4

[B27] Gaines S. (2001). Sex, Love and Science. Nature 413, 255. 10.1038/35095130 11565008

[B28] Genus A. Stirling A. (2018). Collingridge and the Dilemma of Control: Towards Responsible and Accountable Innovation. Res. Pol. 47, 61–69. 10.1016/j.respol.2017.09.012

[B29] Gibbons M. (1999). Science's New Social Contract with Society. Nature 402, C81–C84. 10.1038/35011576 10591229

[B30] Gläser J. Schimank U. (2014). “Autonomie als Resistenz gegen Beeinflussung: Forschungshandeln im organisatorischen und politischen Kontext,,” in Autonomie revisited: Beiträge zu einem umstrittenen Grundbegriff in Wissenschaft, Kunst und Politik. Editors Franzen M. Jung A. Kaldewey D. Korte J. (Weinheim: Beltz Juventa), 45–65.

[B31] Gläser J. Ash M. G. Bunstorf G. Hopf D. Hubenschmid L. Janßen M. (2020). The Independence of Research – a Review of Disciplinary Perspectives and Outline of Interdisciplinary Prospects. Berlin: Technical University of Berlin.

[B32] Goodman A. (2010). Intuition. London: Atlantic Books. 10.21236/ada518604

[B33] Hackett E. J. (2014). Academic Capitalism. Sci. Technol. Hum. Values 39, 635–638. 10.1177/0162243914540219

[B34] Haynes R. D. (2016a). Bringing Science into Fiction. Z. für Anglistik Amerikanistik 64, 127–148. 10.1515/zaa-2016-0015

[B35] Haynes R. D. (2016b). Whatever Happened to the ‘mad, Bad' Scientist? Overturning the Stereotype. Public Underst Sci. 25, 31–44. 10.1177/0963662514535689 24916194

[B36] Herndon L. (2010). Science, Meet Poetry. Cell 143, 1039. 10.1016/j.cell.2010.12.006

[B37] Hilgartner S. (2017). Reordering Life: Knowledge and Control in the Genomics Revolution. Cambridge, Massachusetts: The MIT Press. 10.7551/mitpress/10481.001.0001

[B38] Jarvis M. (2017). AAAS Statement on Scientific Freedom and Responsibility. Science 358, 462. 10.1126/science.358.6362.462-b10.1126/science.358.6362.462-a

[B39] Jasanoff S. (2004). “The Idiom of Co-production,” in States of Knowledge: The Co-production of Science and Social Order. Editor Jasanoff S. (London: Routledge), 1–12.

[B40] Jessop B. (2018). On Academic Capitalism. Crit. Pol. Stud. 12, 104–109. 10.1080/19460171.2017.1403342

[B41] Kaldewey D. (2013). Wahrheit und Nützlichkeit: Selbstbeschreibungen der Wissenschaft zwischen Autonomie und gesellschaftlicher Relevanz. Bielefeld: Transcript. 10.14361/transcript.9783839425657

[B42] Kalleberg R. (2015). “Scientific Misconduct, Plagiarism, and Institutional Control of Misconduct,” in International Encyclopedia of the Social & Behavioral Sciences. Editor Wright J. D. (Amsterdam: Elsevier), 21, 313–317. 10.1016/b978-0-08-097086-8.03022-1

[B43] Kalter F. (2015). “Action, Theories of Social,” in International Encyclopedia of the Social & Behavioral Sciences. Editor Wright J. D. (Amsterdam: Elsevier), 1, 75–79. 10.1016/b978-0-08-097086-8.32002-5

[B44] Kant I. (2011). Groundwork of the Metaphysics of Morals: A German-English Edition. Cambridge: Cambridge University Press. 10.1017/cbo9780511973741

[B45] Kirchhofer A. Roxburgh N. (2016). The Scientist as 'Problematic Individual' in Contemporary Anglophone Fiction. Z. für Anglistik Amerikanistik 64, 149–168. 10.1515/zaa-2016-0016

[B46] Knorr-Cetina K. (1999). Epistemic Cultures: How the Sciences Make Knowledge. Cambridge, Massachusetts: Harvard University Press.

[B47] Koschorke A. (2018). Fact and Fiction: Elements of a General Theory of Narrative. Berlin, Bonn: De Gruyter. 10.1515/9783110349689

[B48] Kuzmics H. Mozetič G. (2003). Literatur als Soziologie: Zum Verhältnis von literarischer und gesellschaftlicher Wirklichkeit. Konstanz: UVK.

[B49] Lepenies W. (1992). Between Literature and Science: The Rise of Sociology. Cambridge: Cambridge University Press.

[B50] Longhito S. (2007). No Lab Is an Island: A Review of Intuition by AllegraGoodman (2006), Dial Press. FASEB j. 21, 2272. 10.1096/fj.07-0802ufm

[B51] Longo M. (2015). Fiction and Social Reality: Literature and Narrative as Sociological Resources. Burlington, Vermont, Farnham: Surrey Ashgate.

[B52] Luhmann N. (2013). Theory of Society, Vol. 2. Stanford, California: Stanford University Press.

[B53] Matthies A. (2016). Spielbälle: Neuverhandlungen der Arbeitswelt im Medium Literatur. Köln: Herbert von Halem Verlag.

[B54] Merton R. K. (1936). The Unanticipated Consequences of Purposive Social Action. Am. Sociological Rev. 1, 894–904. 10.2307/2084615

[B55] Merton R. K. (1973). “The Normative Structure of Science,” in The Sociology of Science: Theoretical and Empirical Investigations. Editor Storer N. W. (Chicago, Illinois: University of Chicago Press), 267–278.

[B56] Mirowski P. Sent E.-M. (2008). “The Commercialization of Science and the Response of STS,,” in The Handbook of Science and Technology Studies. Editor Hackett E. J. (Cambridge: The MIT Press), 635–689.

[B57] Münch R. (1986). Die Kultur der Moderne. Band 1: Ihre Grundlagen und ihre Entwicklung in England und Amerika. Frankfurt am Main: Suhrkamp.

[B58] Münch R. (2014). Academic Capitalism. Universities in the Global Struggle for Excellence. London, New York: Routledge. 10.4324/9780203768761

[B59] Offe C. (1989). “Bindung, Fessel, Bremse: Die Unübersichtlichkeit von Selbstbeschränkungsformeln,,” in Zwischenbetrachtungen: Im Prozeß der Aufklärung. Jürgen Habermas zum 60. Geburtstag. Editors Honneth A. McCarthy T. Offe C. Wellmer A. (Frankfurt am Main: Suhrkamp), 739–774.

[B60] Panofsky A. L. (2010). “7 A Critical Reconsideration of the Ethos and Autonomy of Science,” in Robert K. Merton: Sociology of Science and Sociology as Science. Editor Calhoun C. J. (New York: Columbia University Press), 140–163. 10.7312/calh15112-007

[B61] Parsons T. Platt G. M. (1973). The American University. Cambridge, Massachusetts: Harvard University Press. 10.4159/harvard.9780674423626

[B62] Pilkington O. A. (2017). Popular Science versus Lab Lit: Differently Depicting Scientific Apparatus. Sci. as Cult. 26, 285–306. 10.1080/09505431.2016.1255722

[B63] Pilkington O. A. (2019). “Introduction: What's in a Name?,” in Lab Lit: Exploring Literary and Cultural Representations of Science. Editors Pilkington O. A. Pilkington A. G. (Lanham, Maryland: Lexington Books), 1–12.

[B64] Polanyi M. (1945). The Autonomy of Science. The Scientific Monthly 60, 141–150.

[B65] Renn J. (2020). The Evolution of Knowledge: Rethinking Science for the Anthropocene. Princeton, Oxford: Princeton University Press. 10.2307/j.ctvdf0kpk

[B66] Resnik D. B. (2009). Playing Politics with Science: Balancing Scientific Independence and Government Oversight. Oxford: Oxford University Press. 10.1093/acprof:oso/9780195375893.001.0001

[B67] Robinson K. S. (2004). Forty Signs of Rain. New York: Bantam Books.

[B68] Rohn J. (2008). Abstractions. Nature 451, ix. 10.1038/7175ixb

[B69] Rohn J. (2010a). More Lab in the Library. Nature 465, 552. 10.1038/465552a

[B70] Rohn J. (2010b). The Honest Look. New York: Cold Spring Harbor Laboratory Press.

[B71] Rosa H. (2020). The Uncontrollability of the World. Cambridge, UK, Medford, MA: Polity Press.

[B72] Roxburgh N. (2015). Representing Public Credit: Credible Commitment, Fiction, and the Rise of the Financial Subject. Abingdon, Oxon: Taylor & Francis. 10.4324/9781315646398

[B73] Schaffeld N. (2016). Aspects of the Science Novel. Z. für Anglistik Amerikanistik 64, 121–125. 10.1515/zaa-2016-0014

[B74] Schimank U. Volkmann U. (2017). Das Regime der Konkurrenz: Gesellschaftliche Ökonomisierungsdynamiken heute. Weinheim, Basel: Beltz Juventa.

[B75] Schimank U. (2014). “Planung versus Evolution: Wie verändert sich das Soziale?,” in Handbuch der Soziologie. Editors Lamla J. Laux H. Rosa H. Strecker D. (Konstanz: UTB), 116–130.

[B76] Schimank U. (2015a). “Differentiation: Social,” in International Encyclopedia of the Social & Behavioral Sciences. Editor Wright J. D. (Amsterdam: Elsevier), 6, 391–394. 10.1016/b978-0-08-097086-8.32040-2

[B77] Schimank U. (2015b). Modernity as a Functionally Differentiated Capitalist Society. Eur. J. Soc. Theor. 18, 413–430. 10.1177/1368431014543618

[B78] Schimank U. (2016). Handeln und Strukturen: Einführung in die akteurtheoretische Soziologie. Weinheim, München: Beltz Juventa.

[B79] Schimank U. (2021). “Economization of Science: Insights from Science Novels,” in Under the Literary Microscope: Science and Society in the Contemporary Novel. Editors Farzin S. Gaines S. M. Haynes R. D. (University Park, Pennsylvania: Penn State University Press), 148–172. 10.5325/j.ctv1mvw8k2.11

[B80] Schomberg R. von. (2013). “A Vision of Responsible Research and Innovation,” in Responsible Innovation: Managing the Responsible Emergence of Science and Innovation in Society. Editors Owen R. Bessant J. Heintz M. (Chichester: Wiley), 51–74.

[B81] Schreier M. (2017). Qualitative Content Analysis in Practice. London: SAGE.

[B82] Sennett R. (1999). The Corrosion of Character: The Personal Consequences of Work in the New Capitalism. New York City, New York: Norton.

[B83] Sevänen E. (2018). Modern Literature as a Form of Discourse and Knowledge of Society. Sociologias 20, 48–85. 10.1590/15174522-020004803

[B84] Sismondo S. (2021). Epistemic Corruption, the Pharmaceutical Industry, and the Body of Medical Science. Front. Res. Metr. Anal. 6, 614013. 10.3389/frma.2021.614013 33870067 PMC8028448

[B85] Stehr N. (2015). “Knowledge Society, History of,” in International Encyclopedia of the Social & Behavioral Sciences. Editor Wright J. D. (Amsterdam: Elsevier), 13, 105–110. 10.1016/b978-0-08-097086-8.03160-3

[B86] Stephan P. E. (1996). The Economics of Science. J. Econ. Lit. 34, 1199–1235.

[B87] Stilgoe J. Guston D. H. (2017). “Responsible Research and Innovation,,” in The Handbook of Science and Technology Studies. Editors Felt U. Fouché R. Miller C. A. Smith-Doerr L. (Cambridge, Massachusetts: The MIT Press), 853–880.

[B88] Thomas C. (2006). Getting Away with it. Nat. Med. 12, 1235. 10.1038/nm1106-1235

[B89] Váňa J. (2020). Theorizing the Social through Literary Fiction: For a New Sociology of Literature. Cult. Sociol. 14, 180–200.

[B90] Vogl J. (2014). The Specter of Capital. Stanford, California: Stanford University Press.

[B91] Wagner P. (2008). Modernity as Experience and Interpretation. Cambridge, Malden: Polity.

[B92] Wagner P. (2012). Modernity: Understanding the Present. Cambridge, Malden: Polity.

[B93] Wagner P. (2016). Progress: A Reconstruction. Cambridge, UK, Malden, Massachusetts: Polity.

[B94] Whitley R. (2014). “How Do Institutional Changes Affect Scientific Innovations? the Effects of Shifts in Authority Relationships, Protected Space, and Flexibility,” in Organizational Transformation and Scientific Change: The Impact of Institutional Restructuring on Universities and Intellectual Innovation. Editors Whitley R. Gläser J. (Bingley: Emerald Group Publishing Limited), 367–406. 10.1108/s0733-558x_2014_0000042012

[B95] Wilholt T. Glimell H. (2011). “Conditions of Science: The Three-Way Tension of Freedom, Accountability and Utility,” in Science in the Context of Application. Editors Carrier M. Nordmann A. (Dordrecht: Springer Science+Business Media B.V), 351–370. 10.1007/978-90-481-9051-5_21

[B96] Wilholt T. (2012). Die Freiheit der Forschung: Begründungen und Begrenzungen. Berlin: Suhrkamp.

[B97] Znaniecki F. (1934). The Method of Sociology. New York City, New York: Rinehart & Company.

[B98] Znaniecki F. (1952). Cultural Sciences: Their Origin and Development. Champaign, Illinois: University of Illinois Press.

[B99] Zwart H. (2017). Tales of Research Misconduct: A Lacanian Diagnostics of Integrity Challenges in Science Novels. Cham: Springer International Publishing. 10.1007/978-3-319-65554-3

